# Network construction of aberrantly expressed miRNAs and their target mRNAs in ventricular myocardium with ischemia–reperfusion arrhythmias

**DOI:** 10.1186/s13019-020-01262-4

**Published:** 2020-08-12

**Authors:** Jian Tang, Hong Gao, Yanqiu Liu, Jing Song, Yurong Feng, Guilong Wang, Youqin He

**Affiliations:** 1 Department of Anesthesiology, Guizhou Medical University, Guiyang, Guizhou 550000 China; 2 The Third Affiliated Hospital of Guizhou Medical University, Duyun, Guizhou 558000 China; 3 Department of Anesthesiology, The Affiliated Hospital of Guizhou Medical University, Guiyang, Guizhou 550000 China; 4 Department of Anesthesiology, Guizhou Provincial Tumor Hospital, Guiyang, Guizhou 550000 China

**Keywords:** Reperfusion arrhythmias, microRNAs, mRNA, Bioinformatics

## Abstract

**Background:**

Hypothermic ischemia-reperfusion arrhythmia remains the main factor affecting cardiac resuscitation under cardiopulmonary bypass. Existing research shows that certain miRNAs exhibit significantly different expressions and effects in arrhythmias, however, the effect of miRNAs on the progression of hypothermic ischemic–reperfusion arrhythmias (RA) and its potential mechanism remain to be further explored.

**Methods:**

Sprague-Dawley (SD) rats were randomly divided into two groups (*n* = 8): a normal control group (Group C) and a hypothermic ischemia-reperfusion group (Group IR), which were used to establish a Langendorff isolated cardiac perfusion model. According to the arrhythmia scoring system, rats in group IR were divided into a high-risk group (IR-H) and a low-risk group (IR-L). miRNAs expression profiles of ventricular myocardium with global hypothermic ischemia–reperfusion and those of ventricular myocardium with hypothermic ischemia–RA were established through high-throughput sequencing. Furthermore, the aberrantly expressed miRNAs in myocardium with and without hypothermic ischemia–RA were screened and verified. The target genes of these aberrantly expressed miRNAs were predicted using RNAhybrid and MiRanda software. Based on Gene Ontology (GO) and the Kyoto Encyclopedia of Genes and Genomes (KEGG) databases, we determined the mRNA targets associated with these miRNAs and studied the miRNA–mRNA interaction during the cardiovascular disease progression. The aberrantly expressed miRNAs related to hypothermic ischemia–RA were validated by Real-time Quantitative polymerase chain reaction (RT-qPCR).

**Results:**

Eight significantly aberrantly expressed miRNAs (rno-miR-122-5p, rno-miR-429, novel_miR-1, novel_miR-16, novel_miR-17, novel_miR-19, novel_miR-30, and novel_miR-43) were identified, among which six were up-regulated and two were down-regulated. Moreover, target genes and signaling pathways associated with these aberrantly expressed miRNAs were predicted and analyzed. The miRNA–mRNA interaction network graph showed that GJA1 gene was considered as the target of novel_miR-17.

**Conclusions:**

Aberrantly expressed miRNAs were possibly associated with the formation mechanism of hypothermic ischemia–RA. Specific miRNAs, such as novel_miR-17 and rno-miR-429 are probably new potential targets for further functional studies of hypothermic ischemia–RA.

## Background

Reperfusion arrhythmia (RA) refers to arrhythmia induced by recovering myocardial perfusion after occurrence of coronary occlusion or blockage of myocardial blood flows. RA is one of characteristics of myocardial ischemia–reperfusion injury (MIRI). It mainly appears as various ventricular arrhythmias including ventricular premature beat, ventricular tachycardia (VT), and ventricular fibrillation. RA can even trigger hemodynamic disorder to cause sudden cardiac death (SCD) [1–3]. With the development of various technologies such as anesthesia and cardiopulmonary bypass (CPB), the effect of cardiac surgical procedures has been greatly improved, however, RA remains the major complication during heart resuscitation through open heart surgery under CPB, which directly influences whether the surgery can be successfully conducted or not and patient prognosis. Therefore, investigating the mechanism of formation of RA after hypothermic ischemia is important when trying to prevent these kinds of complications and also provides new targets and directions for clinical treatment.

MicroRNAs (miRNAs) play a crucial role in pathogenesis and progression of various cardiac diseases. They lead to the development of certain cardiac diseases by regulating associated target genes [4]. Multiple miRNAs are shown to participate in the reconstruction of electrophysiology and ion channels by regulating gene expressions of cardiomyocyte during arrhythmias [5]. Clinical research showed that miRNA-1 in patients’ serum increases after conducting CPB [6]. Moreover, Bostjancic et al. [7, 8] validated two target genes (GJA1 and KCNJ2) from the nucleotide sequence at the 5′ end of miRNA-1, which are coded as Cx43 and potassium channel subunits Kir2.1, respectively. The over-expression of mi-RNA-1 can inhibit the expressions of GJA1 and KCNJ2, resulting in the electrolyte disorder of cardiomyocyte thus triggering arrhythmias. In hypothermic ischemia–RA, whether there are miRNAs affecting RA by regulating GJA1 and KCNJ2 or not is unknown.

The gap junction (GJ), as the basic cardiac electrophysiological structure, can sustain normal coupling in electrocardiogram (ECG) signals and mechanical coupling by mediating intercellular electrochemical communication. Connexin (Cx) is the basic unit of GJ [9], and its normal expression is crucial for guaranteeing normal electric coupling and conduction between cardiomyocytes. Cx43 is subjected to lateralization and dephosphorylation during ischemia–reperfusion injury, thus leading to lateral conduction, formation of re-entry and electrical uncoupling thus triggering RA [10, 11]. By observing myocardium with ischemia–RA, we found that the expression, distribution, and phosphatization states of Cx43 are all changed to different extents to reduce the conduction velocity of myocardium and increase the probability of formation of RA [12]; however, the specific correlation between Cx43 and miRNAs in hypothermic ischemia–RA has not been clarified.

In summary, there is no systematic study available on how microRNAs differentially express in RA myocardium after hypothermic ischemia-reperfusion and how to regulate RA. Therefore, we attempt to establish an isolated rat heart model of hypothermic ischemia-reperfusion. The miRNAs related to hypothermic ischemia–RA were screened by high-throughput sequencing. By using bioinformatics analysis methods, including the Gene Ontology (GO) and Kyoto Encyclopedia of Genes and Genomes (KEGG) databases, the miRNA–mRNA interaction was further analyzed. On this basis, the potential functions of aberrantly expressed miRNAs and the possible mechanism of interaction between the miRNAs and hypothermic ischemia–RA through genes were determined. This finding is expected to provide new targets and directions for preventing hypothermic ischemia–RA.

## Methods

### Preparation of isolated rat heart models

Sixteen healthy male Sprague-Dawley (SD) rats of 2 to 3 months old, weighing 300 to 400 g at clean grade were provided by the Experimental Animal Center, Guizhou Medical University (Guiyang, Guizhou Province, China). Heparin (3%, batch number: 51606118, Jiangsu Wanbang Biochemical Medicine Co., Ltd) of 500 Iu/kg was intraperitoneally injected for anticoagulation. After administration for 10 min, 40 mg/kg of pentobarbital was intraperitoneally injected for anesthesia. The chest of the rats was opened (immediately after the anesthesia took effect) to remove the heart, which was then placed in the Krebs-Henseleit (K-H) solution at 4 °C before trimming and exposing the aorta. The aorta was fixed on Langendroff perfusion equipment (Shanghai Alcott Biotech Co., Ltd) to conduct acyclic retrograde perfusion at constant temperature (37 °C) and constant pressure (8.65 kPa) with a K-H solution saturated with 95% O_2_ and 5% CO_2_. The model for Langendroff perfusion of isolated rat hearts was regarded as having been successfully prepared if the heart rhythm (HR) was recovered within 3 min after balance perfusion and HR was greater than 180 times/min at the end of balance perfusion (Krebs-Henseleit (K-H) buffer solutions included: NaCl 118 mmol/L, MgSO_4_ 1.2 mmol/L, KCl 4.7 mmol/L, KH_2_PO_4_ 1.2 mmol/L, and NaHCO_3_ 25 mmol/L, glucose 11 mmol/L, and HEPES 10 mmol/L, pH 7.35, adjusted with NaOH).

### Experimental treatments

Sixteen prepared models for Langendroff perfusion of isolated rat hearts were divided randomly into two groups (*n* = 8 in each group): a control group (Group C) and an ischemia reperfusion (IR) group. The former was subjected to continuous perfusion for 120 min with K–H solution at 37 °C. As for the latter, after balance perfusion with K–H solution at 37 °C for 30 min, St. Thomas solution (St. Thomas’ Hospital cardioplegic solution, 20 ml/kg) at 4 °C was injected at the root of the aorta to allow 60 min of cardiac arrest. The periphery of the heart was protected with K–H solution at 4 °C. Then, St. Thomas solution (10 ml/kg) at 4 °C was perfused again after cardiac arrest had lasted for 30 min and K–H solution at 37 °C was re-perfused for 30 min after 60 min of cardiac arrest (St. Thomas’ solution included: NaCl 110 mmol/L, CaCl 1.2 mmol/L, MgCl_2_ 16 mmol/L, KCl 16 mmol/L, and NaHCO_3_ 10 mmol/L at pH 7.6 to 7.8. This reagent comes from the Department of Anesthesiology, Guizhou Medical University, China).

#### Evaluation of arrhythmia

The method used to measure the ECG was as previously described [13]. The electrode wires were connected to the signal input wires of the BL-420F biological function experiment system (Chengdu Taimeng Software Co., Ltd) respectively. The ECG was amplified and analyzed using a BL-420 biological function system. Bipolar electrograms between the atrial and ventricular electrodes were used to monitor arrhythmias [14].

The severity of arrhythmia during reperfusion that occurred in each individual heart was assessed via the Curtis and Walker scoring system as well as the Lepran scoring system [15]. A discrete and identifiable premature QRS complex was diagnosed as premature ventricular contractions (PVCs). A run of four or more PVCs was identified as VT. VF was defined as a ventricular rhythm without a recognizable QRS complex and thus an immeasurable HR.

No arrhythmia was observed in two IR rats (score 0) during reperfusion, but different types of arrhythmia can be observed in the other six rats of the IR group. One of the six rats was found with ventricular premature beats (91 PVC, score 2) and returned to normal rhythm after 1.63 min. The atrioventricular block was observed in three of the six rats and this lasted for 1.25 min (score 3), 11.7 min (score 5), and 21.83 min (score 5) respectively before returning to normal rhythm. Ventricular fibrillation accompanied by premature ventricular beats could be observed in the two remaining rats and this was maintained for 11.5 min (score 5) and 5.42 min (score 5) respectively before normal rhythm was restored. According to the two arrhythmia scoring systems, four IR rats with arrhythmia score > 3 were placed in a high risk group (IR-H), and the other IR rats with arrhythmia score ≤ 3 were placed in a low risk group (IR-L).

### Experimental grouping

Rat hearts with scores higher, or not higher, than 3 were graded as having a high risk of IR (IR-H) or low risk of IR (IR-L), IR-H group+ IR-L was collectively called the ischemia-reperfusion (RA) group. Then, the hearts were divided into three groups based on the degree of arrhythmia: a Group C, Group IR-L, and Group IR-H, each containing specimens of ventricular myocardium of four rats. Myocardium of the left ventricle was removed immediately after perfusion and the hearts were frozen and transferred to a freezer to be stored at − 80 °C.

### High-throughput sequencing and analysis of aberrant expression of miRNAs

The Trizol method was employed to extract the total RNA of the specimens and the RNA was treated with DNase I to eliminate DNA pollution. All these procedures followed published instructions. Using a micro-nucleic acid protein tester (Agilent Company), formaldehyde denaturing gradient gel electrophoresis (DGGE), and capillary electrophoresis, the extracted total RNA was tested to ensure that its concentration and completeness reached the requirements for sequencing. The Beijing Genomics Institute was entrusted to perform high-throughput sequencing for the aforementioned samples on the BGISEQ-500 platform. The miRNA expression of the samples was standardized as transcripts per million (TPM). For given transcripts, the gene expression was estimated by aligning the number of fragments in a gene region. The DEGseq [16–18] method was used to reveal changes in the expression of the gene transcripts of the three groups of samples. After obtaining the value of *P*, multiple hypothesis tests were conducted and the corrected *P* was represented by false discovery rate (FDR), satisfying FDR < 0.05. If the fold change in expression was equal to or greater than 2, the gene was deemed to have been aberrantly expressed.

### Prediction of target genes of aberrantly expressed miRNAs

RNAhybrid and miRanda [17] databases were used to predict the target genes of aberrantly expressed miRNAs and those genes predicted mapped to each term in the GO database to count the number of genes that were mapped to each term [19]. Then, the *P*-value was corrected by using the Bonferroni method, a corrected *P*-value ≤ 0.05 was taken as a threshold. GO terms fulfilling this condition were defined as significantly enriched GO terms. Then, by using KEGG database (http://www.genome.jp/kegg/), target genes of aberrantly expressed miRNAs were mapped and the same calculation method was used to obtain the KEGG analysis results. Finally, the mRNA–miRNA interaction network graph was established.

### Analysis of genes associated with the progression of cardiovascular diseases

All of the target mRNAs of differently regulated miRNAs were screened using RNAhybrid and miRanda. Then, the mRNA–miRNA process was analyzed to build networks of the physiological system and pathophysiology. Based on GO and KEGG cardiac function databases, the mRNAs that participated in pathophysiological process of cardiovascular diseases (CVDs), including hypertrophy, fibrosis, conduction abnormality, and arrhythmia, were selected.

### Screening of the target miRNAs

Based on gene function analysis, KEGG pathway analysis, and mRNA–miRNA interaction, bioinformatics analysis was conducted on transcripts of miRNAs related to KCNJ2/GIA1 genes. The target miRNAs regulating expressions of KCNJ2 or GJA1 in the hypothermia ischemic–RA were preliminarily screened.

### Reverse transcription-quantitative PCR

RT-qPCR verification was performed as described elsewhere [20]. The left ventricular myocardial tissue (apex of the left ventricle) was used to extract total RNA with TRIzol reagent (Invitrogen) according to the manufacturer’s instructions. We performed qPCR on four miRNAs with higher expression level (rno-miR-122-5p, novel_miR-17, rno-miR-429, and novel_miR-19), which were closely related to the biological processes of arrhythmias. The RNAs were reverse-transcribed to cDNA with stem-loop-like RT primers (Biofavor, Wuhan, China) that are specific for only the mature microRNA species and then quantified on an Applied Biosystems 7500 Real-Time PCR system (Applied Biosystems, Carlsbad, CA, USA) using SYBR-Green PCR Master Mix (Vazyme, Nanjing, China). The PCR reaction was carried out at 95 °C for 5 min, followed by incubation at 95 °C for 15 s, 65 °C for 15 s, and 72 °C for 32 s (repeated for 40 cycles). Each PCR was repeated at least three times. The relative expression level of each microRNA was normalized against RNU6B expression levels. The fold-change was calculated according to the 2^-∆∆CT^ method [21].

### Statistical analysis

We conducted hierarchical clustering for differentially expressed miRNAs using pheatmap, a function of R. For clustering more than two groups, we performed the intersection and union DESs between them [15]. Based on “GO Term Finder” (http://www.yeastgenome.org/goTermFinder), significantly enriched GO terms with corrected *p*-value ≤ 0.05 were screened. The differentially expressed transcripts were aligned to the KEGG pathway database (http://www.genome.jp/kegg/) for statistical analysis: we also removed significantly enriched KEGG pathways with corrected *p*-value ≤ 0.05. All data are presented as the mean ± standard deviation (SD). Statistical analysis was performed using SPSS 22.0 software (SPSS, Inc., Chicago, IL, USA). The one-way ANOVA was used for comparisons between two groups: if there were any differences, a post hoc LSD test was conducted (wherein a value of *p* < 0.05 was considered to indicate a statistically significant difference).

## Results

### Aberrant expression of miRNAs

**Fig. 1:**
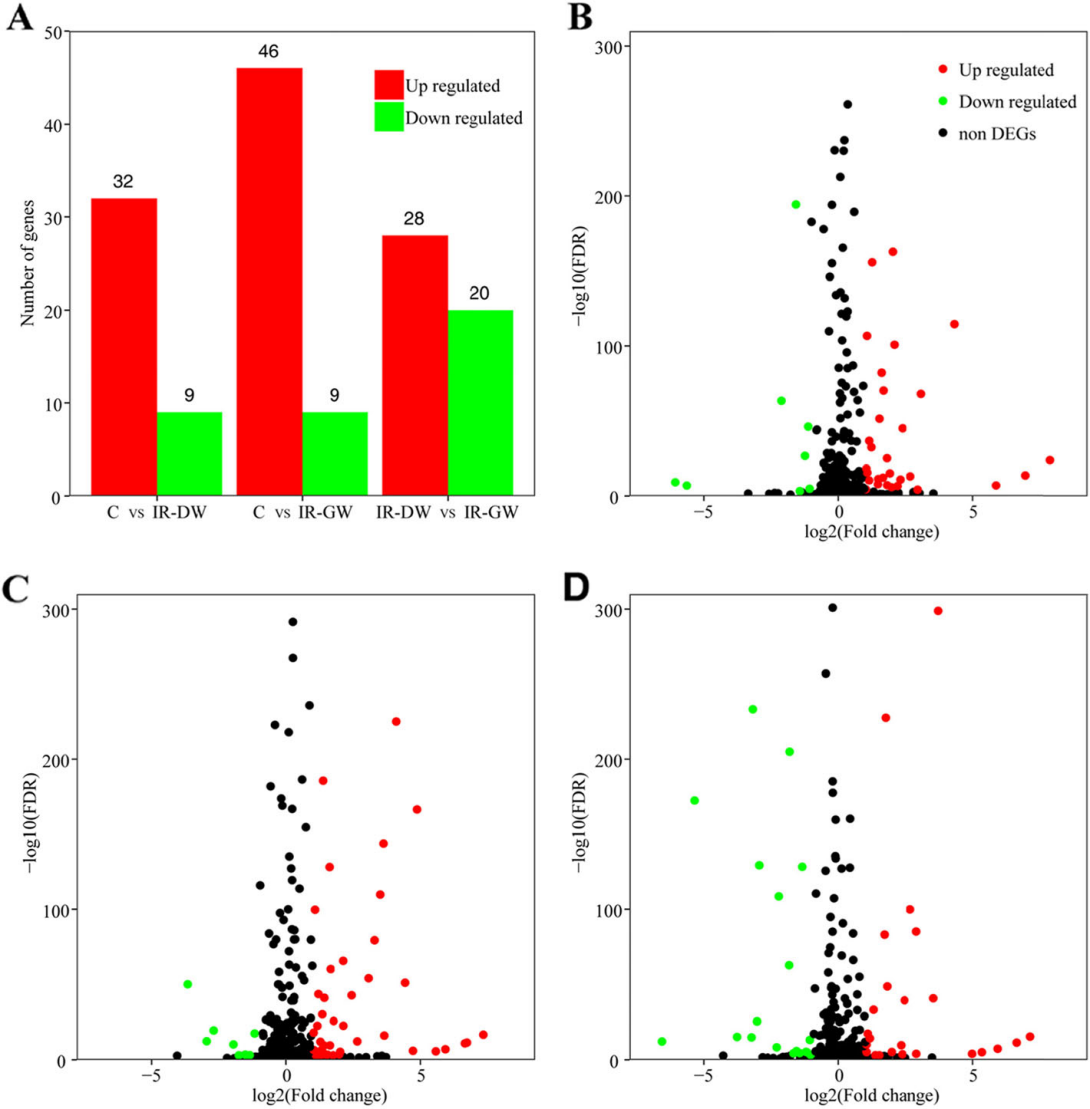
The plots of differentially expressed microRNAs. **a**. Group names are listed at the bottom. The number of the significantly (*p* < 0.01) expressed microRNAs are shown on the left. Significant differential expression of 85 microRNAs: green, downregulation; red, upregulation. **b**.volcano plot of differentially expressed microRNAs from high-throughput sequencing. (C vs IR-DW). The horizontal lines correspond to 2-fold up and down, respectively, and the vertical line represents a FDR. So the red and the green points in the plot represent the differentially expressed microRNAs with significant difference. **c**. volcano plot of C vs IR-GW. **d**. volcano plot of IR-DW vs IR-GW

According to DEGseq method, some 85 significantly aberrantly expressed miRNAs commonly existed in the three groups (Fig. 1a). Among the miRNAs with significant aberrant expressions, expressions of 46 miRNAs were up-regulated in the C and IR-H groups while those of nine others were down-regulated (Fig. 1b). In the C and IR-L groups, 32 miRNAs were found to have up-regulated expressions while nine showed down-regulated expressions (Fig. 1c). As for the IR-H and IR-L groups, 28 miRNAs had up-regulated miRNAs and 20 exhibited down-regulated miRNAs (Fig. 1d).

### Screening of miRNAs associated with hypothermia ischemic–RA

According to the principle of fold change ≥2 and FDR < 0.05, eight miRNAs that are most closely associated with hypothermia ischemic–RA were selected from the 85 aberrantly expressed miRNAs (Table 1). A comparison of expressions of the miRNAs indicated that the three groups had discrepancies in the expressions of novel_miR-1 and novel_miR-16, and IR-H and IR-L groups showed a difference in expressions of novel_miR-16 and novel_miR-30. The RA groups and the C control also displayed differences in rno-miR-122-5p expression. A significant increase in the expression of novel_miR-17 was observed in the IR-H group compared with the IR-L and C groups. Although the expressions of novel_miR-1 and novel_miR-16 did not exhibit significant differences in the three groups, they showed higher expressions in the IR-H group.

**Table 1:** Eight microRNAs with the highest expression among 85 differentially expressed microRNAs

Mature-ID	Fold change	*P* value	Regulation	FDR
novel-miR-1	1.57	0.0000045	up	< 0.001
novel-miR-16	4.32	0.0000562	dpwn	< 0.001
novel-miR-17	2.57	0.0000765	up	< 0.001
novel-miR-19	5.87	0.0000694	up	< 0.001
novel-miR-30	6.63	0.0000425	up	< 0.001
novel-miR-43	7.13	0.0000238	up	< 0.001
rno-miR-122-5p	2.38	0.0000758	up	< 0.001
rno-miR-429	5.34	0.0000346	down	< 0.001

### Predicting target genes of aberrantly expressed miRNAs

RNAhybrid and miRanda were employed to predict target genes of aberrantly expressed miRNAs and it was found that each miRNA had multiple predicted target genes. Totally 2810 target genes were predicted for the eight aberrantly expressed miRNAs. These target genes are probably the targets related to hypothermia ischemic–RA.

### GO and KEGG data analysis

**Fig. 2:**
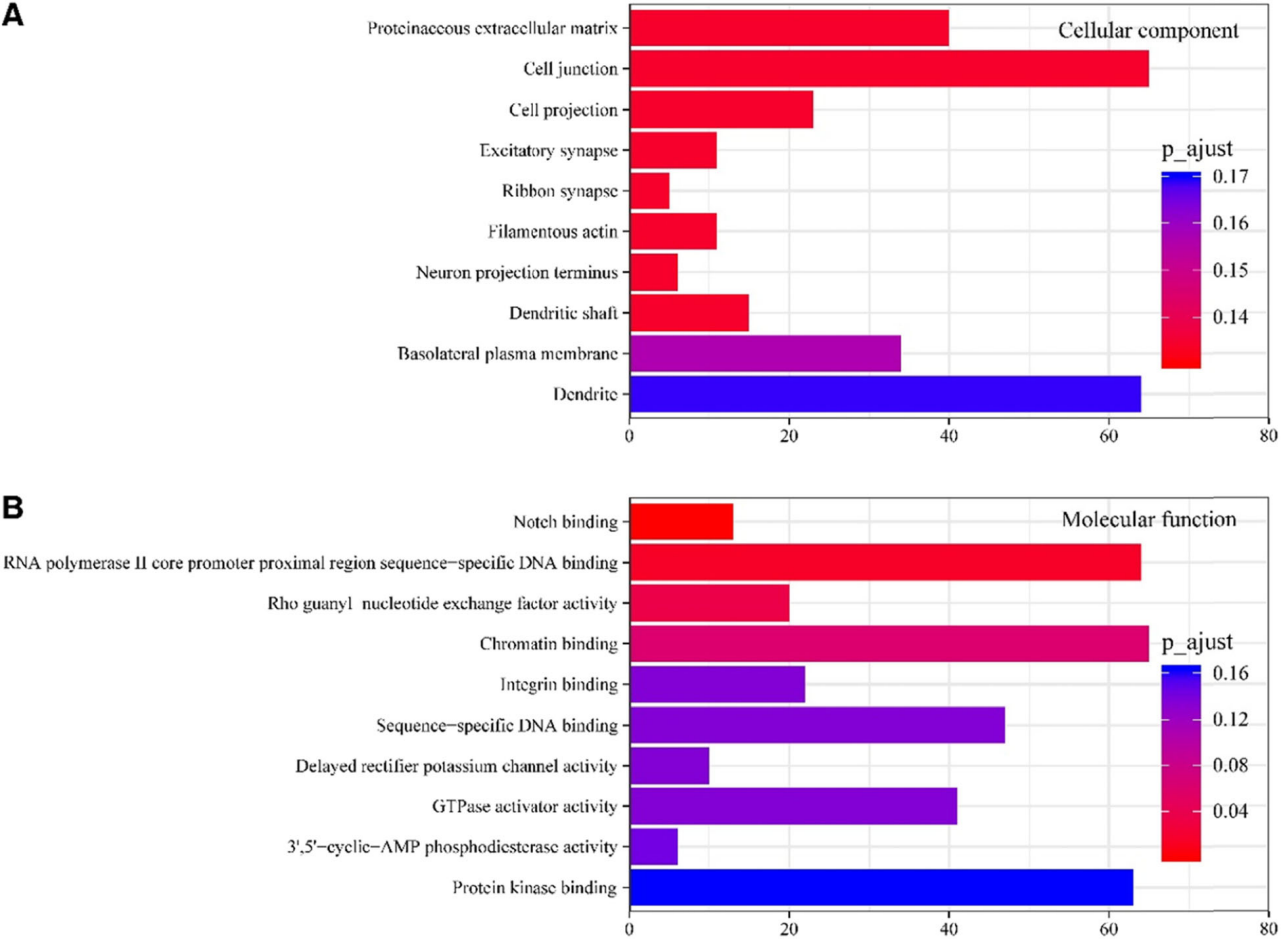
GO Analysis of differentially expressed MicroRNAs. **a**.GO analysis for differentially expressed (DE) mRNACellular component (CC). Bar plot explanation (Enrichment Score): the bar plot shows the top ten Enrichment Score value of the significant enrichment terms. **b**. GO analysis for differentially expressed (DE) mRNA-Molecular function (MF). Bar plot explanation (Enrichment Score): the bar plot shows the top ten Enrichment Score value of the significant enrichment terms

**Fig. 3:**
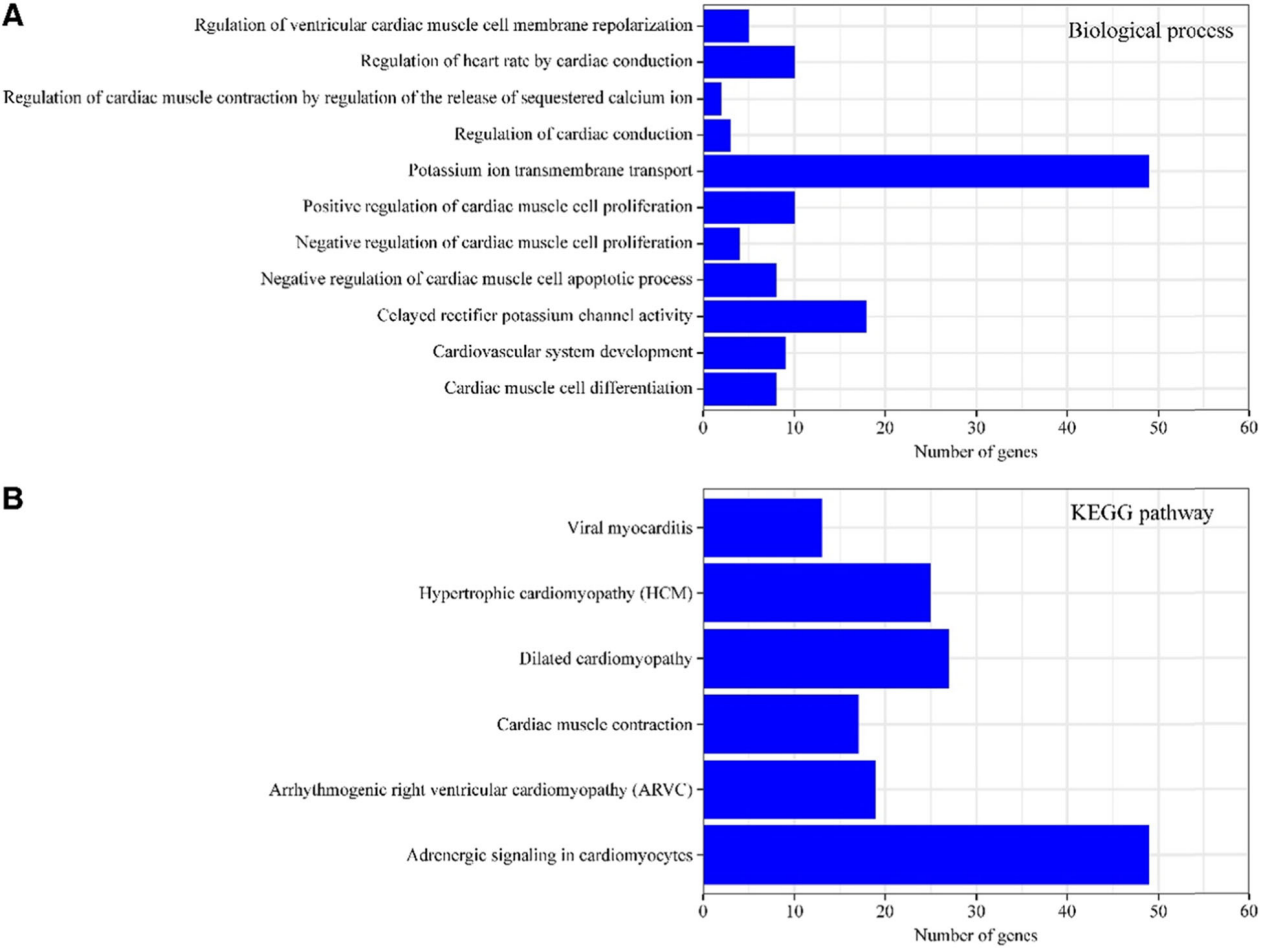
GO Analysis of differentially expressed MicroRNAs. **a**. GO analysis for differentially expressed (DE) mRNA-Biological process (BP). Bar plot explanation (Enrichment Score): the bar plot shows the top eleven Enrichment Score value of the significant enrichment terms. **b**. Pathway analysis of target genes of differentially expressed (DE) microRNAs. Target genes were enriched into different pathways About heart disease based on KEGG pathway database, the top 6 pathways were shown above

Three GOs separately described the biological process (BP), molecular function (MF), and cellular component (CC) of genes. In the current research, aberrantly expressed miRNAs were enriched in numerous CCs, included cell junction, proteinaceous extracellular matrix, cell projection, dendrite, and basolateral plasma membrane (Fig. 2a). Likewise, the molecular function, delayed rectifier potassium channel activity, RNA polymerase II core promoter proximal region sequence−specific DNA binding, protein kinase binding, and chromatin binding were also affected (Fig. 2b). The biological process affected included: potassium ion transport across cell membranes, delayed rectifier potassium channels, and regulation of heart rate by cardiac conduction (Fig. 3a). Then, enrichment of target genes of aberrantly expressed miRNAs in KEGG pathways was further analyzed. The results showed that the target genes participated in six signaling pathways were associated with cardiac diseases (Fig. 3b). These signaling pathways mainly included those of epinephrine, arrhythmogenic right ventricular cardiomyopathy (ARVC), and cardiac hypertrophy.

### Bioinformatics data

**Fig. 4:**
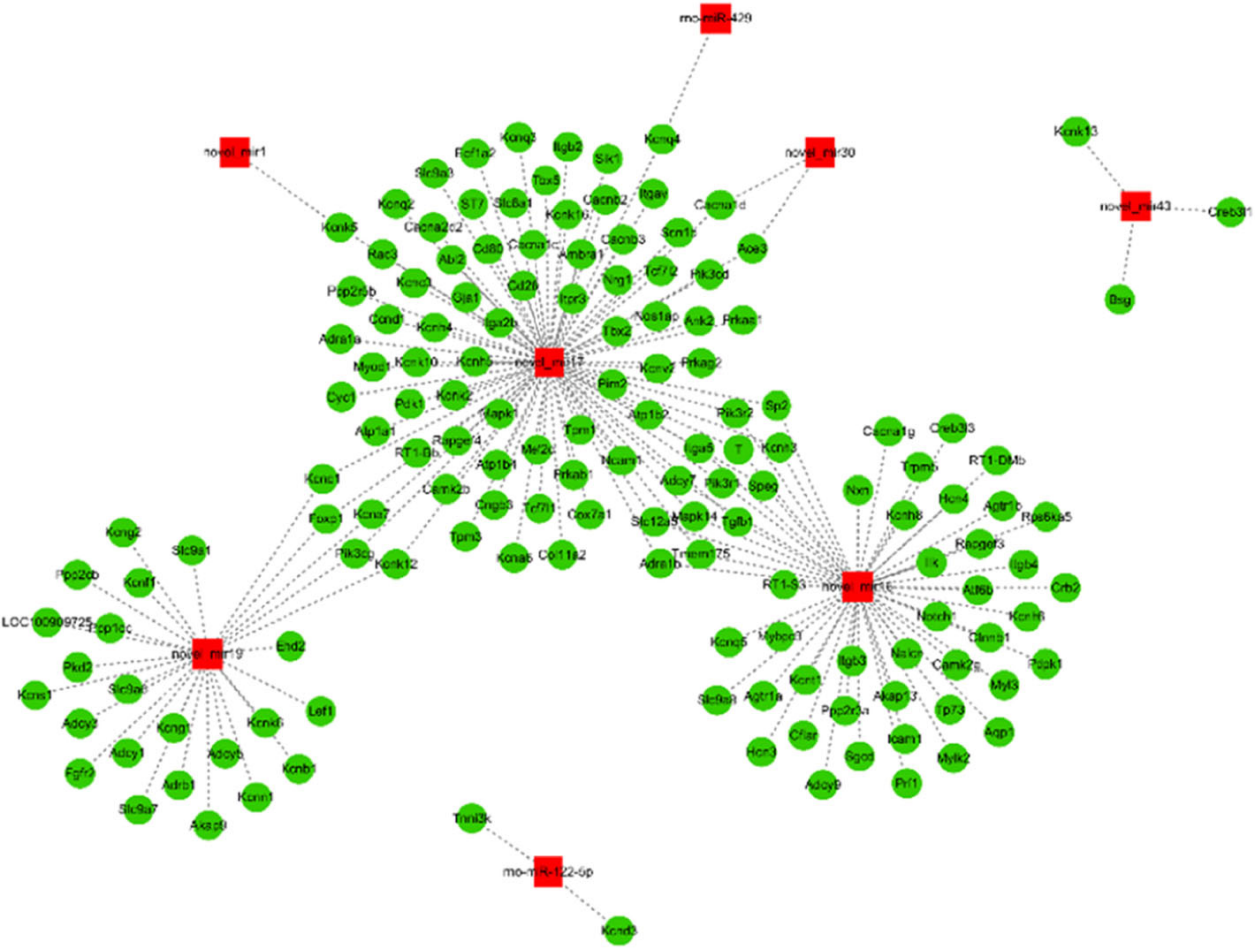
Interaction of miRNAs with mRNAs involved in cardiovascular processes. It was revealed that GJA1 gene was considered as the target of novel-miR-17. Green box - mRNAs; red symbols – miRNA. cytoscape software data

**Fig. 5:**
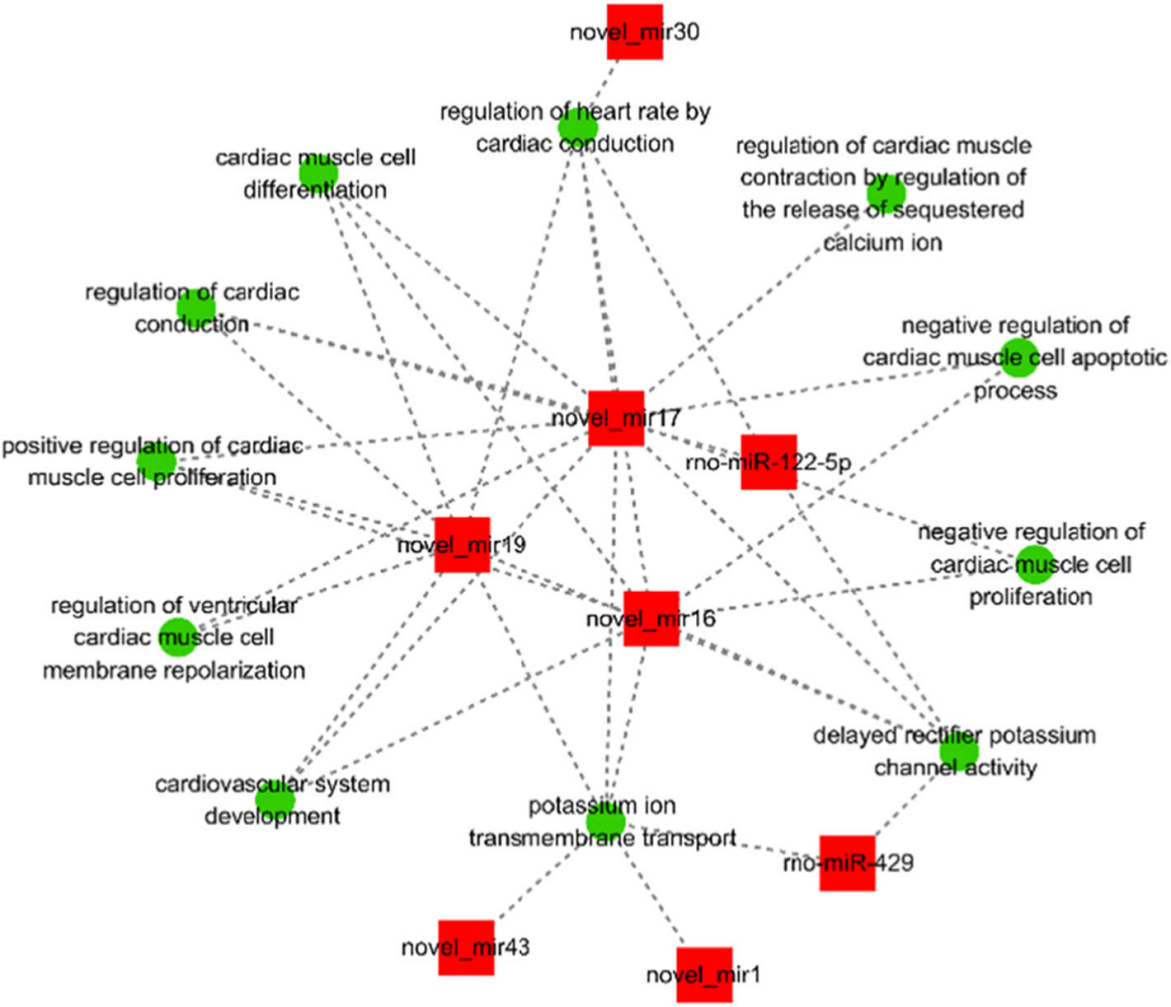
Interaction of miRNAs with pathophysiological processes related to RA. Green Box – cardiac pathophysiological processes; red symbols - miRNAs. cytoscape software data

**Fig. 6:**
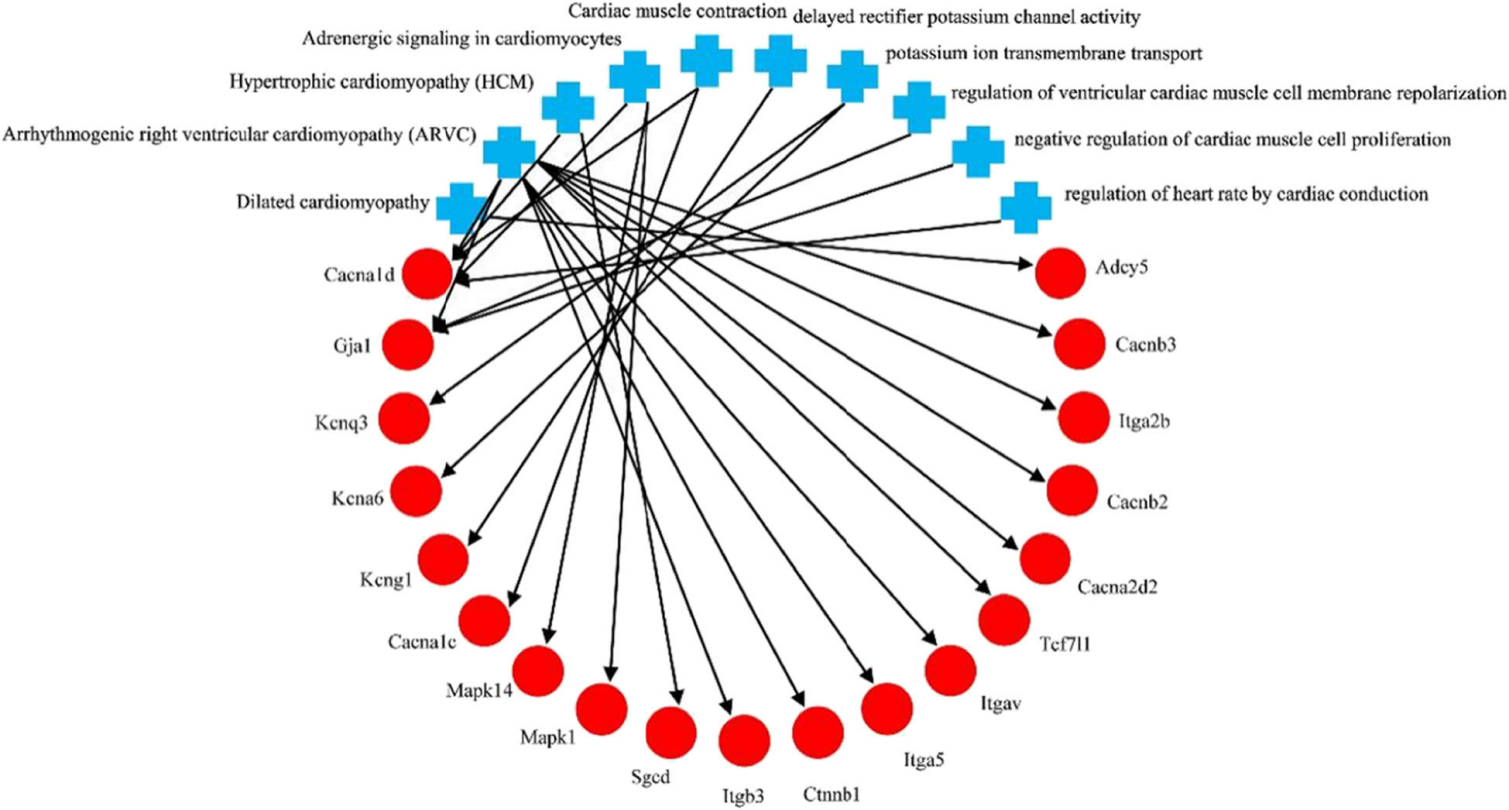
Interaction of mRNAs with pathophysiological processes associated with RA. Red symbols - mRNA; blue symbols- cardiac pathophysiological processes. Cytoscape software data

A total of 151 mRNAs showed miRNA–mRNA interactions (Fig. 4). We observed the correlations of these miRNAs with various cardiac pathophysiological processes and the process that were likely to trigger RA or finally develop to RA (Fig. 5). The relationship between the two analyses showed that 19 of the 151 mRNAs were associated with the development of cardiac diseases (Fig. 6). The target gene GJA1 of novel_miR-17 participated in the proarrhythmia and the associated pathways, which is consistent with previous research results. The target gene KCNQ4 of rno-miR-429 and novel_miR-17 was associated with the delayed rectifier potassium channel and the potassium ion transport across cell membranes. Through screening using the pathway analysis software, no target genes associated with the electrocardiological mechanism regulated by novel_miR-1, novel_miR-13, and novel_miR-43 were found.

### RT-qPCR validation of aberrantly expressed miRNAs

**Fig. 7:**
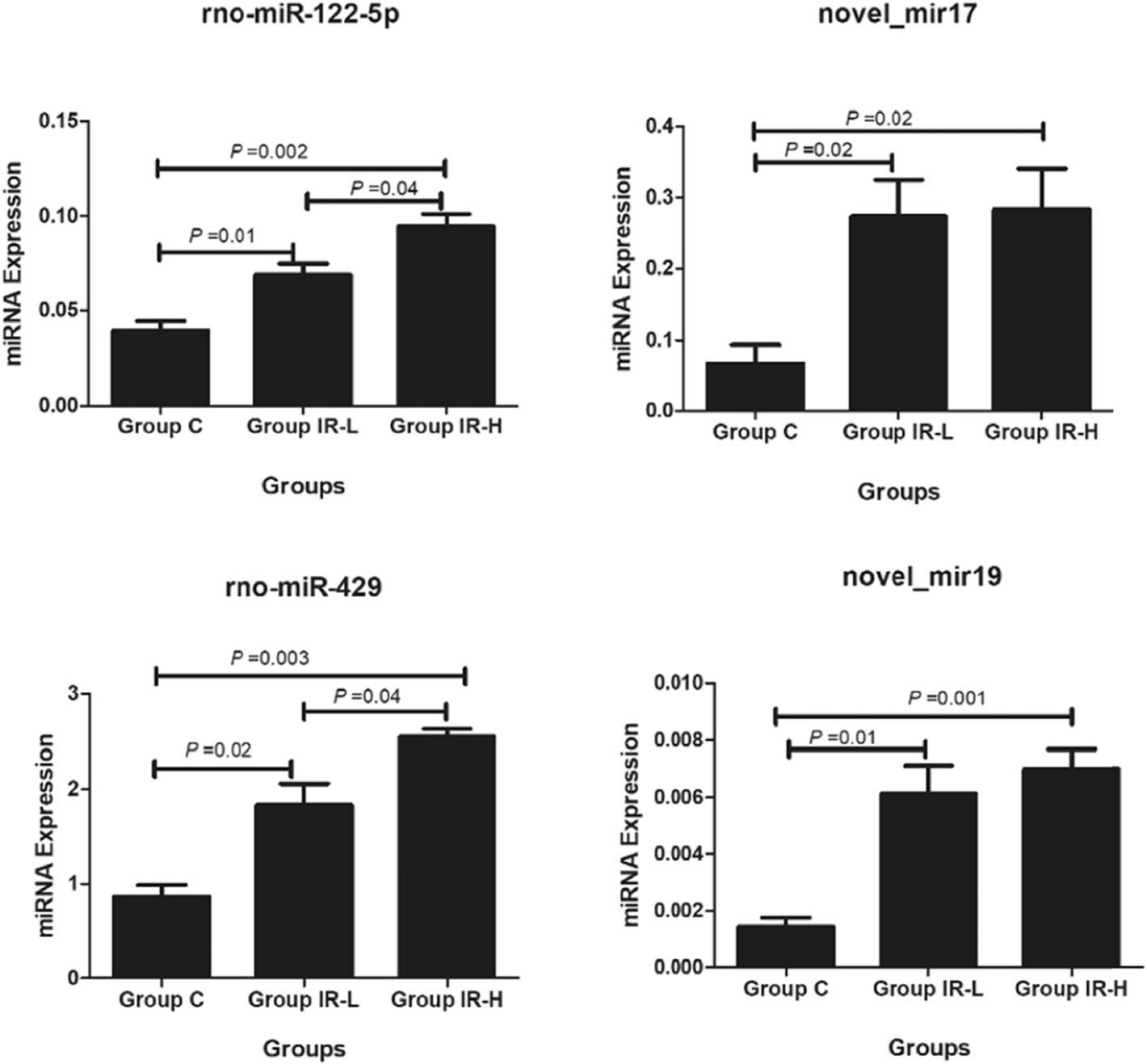
Expression of miRNAs (novel-miR-17, novel-miR-19, rno-miR-429, rno-miR-122-5p) among control, IRDW and IR-GW groups. Group names are listed at the bottom. Relative expression was quantified using the comparative CT

To validate the results of high-throughput sequencing results, we randomly selected four significantly differentially expressed microRNAs to perform RT-qPCR analysis with specific stem-loop RT primers to examine the expression levels of the mature microRNAs, including rno-miR-122-5p, novel_miR-17, rno-miR-429, and novel_miR-19. The results showed that the changes of four abnormally expressed microRNAs in the three groups were in agreement with the high-throughput sequencing results (Fig. 7).

## Discussion

The miRNAs regulate the gene expression by inhibiting the translation process of protein-coding mRNAs through combination with the 3′ untranslated region (3’UTR) of specific mRNAs [22–24]. Research in recent years has shown that miRNA molecules play an essential role in the progression of cardiac diseases such as MIRI and arrhythmia [25, 26]. Human intervention on the expression of some miRNAs affects the progression of RA, the formation mechanism of which is closely associated with electrophysiological reconstruction. The miRNAs are key molecules in arrhythmia, so their intervention and regulation has become a new target for treatment of diseases [27, 28]. The target genes of the four miRNAs newly discovered in the research are mainly found in the ARVC pathway, while effects of these miRNAs in biological and pathological research have not yet been reported. Therefore, it is essential to study whether or not these miRNAs have important effects on the RA by regulating relevant signaling pathways. This result provides more possible directions for research into hypothermia ischemic–RA.

Aberrantly expressed miRNAs and their target protein-coding RNAs consist of a complicated network, which participates in the regulation of the formation and progression of RA. Previous research showed that miRNAs and Cx43 participate in the formation of RA [29], while the correlation mechanism between the two is rarely investigated in detail. Apart from ventricular arrhythmia, upward shift of the S-T segment, and QT prolongation, the ECG also exhibited a prolonged QRS interval after ischemia reperfusion, all of which are associated with a slower intermyocardial conduction velocity after ischemia reperfusion [3]. GJ, as the basic structure of cardiac electrophysiology, maintains the normal coupling in EGC signals and mechanical coupling by mediating intercellular electrochemical communications. The normal expression of Cx43 coded by gene GJA1 is crucial for electric coupling and conduction between cardiomyocytes [30]. We also found that the expression, distribution, and phosphorylation state of Cx43 in the myocardium of ischemic–reperfusion arrhythmia all changed to different extents, thus decreasing the conduction velocity of myocardium and increasing the formation of RA [11, 31]. Therefore, regulating the expression of Cx43 is one of the key factors affecting the formation of hypothermia ischemic–RA. In the research, GJA1 is the target of novel-miR-17. Whether or not novel-miR-17 can regulate Cx43 protein by acting on CJA1 and therefore influence the formation of hypothermia ischemic–RA warrants future research.

Through GO functional and KEGG pathway enrichment analyses, it was found that the target genes of the eight significantly aberrantly expressed miRNAs participated in potassium ion transport across cell membranes and the delayed rectifier potassium channels. Therefore, aberrantly expressed miRNAs are assumed to be capable of regulating the target genes to participate in the mechanism of action of hypothermia ischemic–RA via the above channels. After suffering hypothermic ischemic injury, the electrophysiological changes in irregular myocardium repolarization, including MAPD prolongation and increasing transmitted drug resistance (TDR) in the myocardium, indicated that the potassium channel possibly participated in the formation of RA [32, 33]. According to the regulation network of the aberrantly expressed miRNAs and their mRNAs, the relevant target genes KCNQ4 and KCNA6 of miR-429 and novel-miR-17 were found to be associated with potassium ion transport across cell membranes and delayed rectifier potassium channels. Therefore, they result in the formation of RA by participating in the regulation of the ion channels. The specific regulation mechanism will be an important topic in future research. Moreover, the miRNA–mRNA interaction also indicated that seven mRNAs (CACNA1C, ITGB3, CTNNB1, GJA1, ITGAV, CACNB3, and TCF7L1) are probably the targets of the candidate miRNAs that participate in the progression of CVDs. This provides more possible targets and directions for future research.

### Limitations of the study

The Langendoff isolated perfusion model used in this study is a classical ischemia/reperfusion model of a denervated heart, which can better mimic clinical CPB. After balanced perfusion, the beating heart was regarded as normal. Cardioplegia was used to simulate cardiac arrest after aortic clamping and rebeating after reperfusion. Although we have tried to mimic the clinical setting regarding temperature, application, and mimic the CPB system used during cardiac surgical procedures, our experimental setting in an isolated rat heart model is artificial. Moreover, the reperfusion arrhythmia was created in a beating and perfused heart for 120 min and not a working heart over a longer time. So, the main type of atrioventricular block (AV) is Mobitz-type I second-degree AV block, and other types of atrioventricular block are relatively rare. However, compared with other types of arrhythmias, the occurrence of AV is less and the time is shorter. The total number of animals used was also small. At present, we do not know the specific relationship between the eight significantly differentially expressed microRNAs and reperfusion arrhythmias, which warrants further investigation. Despite these limitations, this model can eliminate the interference of neurological and humoral factors, no other experimental model comes closer to the clinical setting.

## Conclusion

In conclusion, Novel-miR-17 probably has effects on the CJA1 gene, thus regulating the formation of RA. Meanwhile, the biological analysis showed that novel-miR-17 and miR-429 probably regulate their target genes to influence the ion channels and affect the formation of RA. This result indicated that novel-miR-17 is a potential biological marker. We will further study the functions of these miRNAs, so as to broaden our knowledge of the mechanism of action of hypothermia ischemic–RA.

## Data Availability

All raw data are available upon request and the corresponding author, Prof. Hong Gao (E-mail: 2169617@qq.com) should be contacted to request the data.

## Abbreviations

MIRI: Myocardial ischemia–reperfusion injury
VT: Ventricular tachycardia
SCD: Sudden cardiac death
CPB: Cardiopulmonary bypass
GJ: Gap junction
ECG: Electrocardiogram
Cx: Connexin
SD: Sprague-Dawley
K-H: Krebs–Henseleit
DGGE: Denaturing gradient gel electrophoresis
TPM: transcripts per million
FDR: False discovery rate
CVDs: Cardiovascular diseases
BP: Biological process
MF: Molecular function
CC: Cellular component
ARVC: Arrhythmogenic right ventricular cardiomyopathy
M-APD: Monophasic action potential durations
TDR: Transmural dispersion of repolarization
AV: Atrioventricular block
